# Neutrophil-to-Lymphocyte Ratio Correlation With the Synergy Between Percutaneous Coronary Intervention With Taxus and Cardiac Surgery (SYNTAX) Score in Patients With Non-ST-Segment Elevation Acute Coronary Syndrome

**DOI:** 10.7759/cureus.98890

**Published:** 2025-12-10

**Authors:** Mohamed Mabrouk Tohamy, Paula Derias, Dina Hassan Eldahshan, Hesham Boushra Mahmoud

**Affiliations:** 1 Cardiology, Faculty of Medicine, Beni-Suef University, Beni-Suef, EGY; 2 Cardiology, Aswan Heart Centre, Aswan, EGY; 3 Cardiology, University Hospitals Coventry and Warwickshire, Coventry, GBR; 4 Clinical Pathology, Faculty of Medicine, Beni-Suef University, Beni-Suef, EGY

**Keywords:** acute coronary syndrome, angiographic severity, coronary artery disease (cad), ischemic heart disease (ihd), neutrophil to lymphocyte ratio (nlr), syntax score

## Abstract

Background: The neutrophil-to-lymphocyte ratio (NLR) is a simple inflammatory marker that may reflect the extent of coronary atherosclerotic complexity. This study examined the relationship between NLR and angiographic disease severity in individuals presenting with non-ST-segment elevation acute coronary syndrome (NSTE-ACS), using the synergy between percutaneous coronary intervention with taxus and cardiac surgery (SYNTAX) score as an anatomical reference standard.

Methods: In this prospective observational study, 100 consecutive patients with NSTE-ACS underwent coronary angiography at two tertiary centres in Egypt between July 2020 and June 2021. Patients with persistent ST-segment elevation, active infection, chronic inflammatory diseases, malignancy, chronic obstructive pulmonary disease, or chronic steroid use were excluded. A single baseline NLR measurement was obtained at admission before angiography. Laboratory testing at both centres used the same automated analyser. SYNTAX scoring was performed independently by two interventional cardiologists who were blinded to clinical and laboratory data, with discrepancies resolved by consensus. Patients were stratified into three tertiles based on admission NLR: low NLR (≤3.65), intermediate NLR (>3.65 to ≤5.1), and high NLR (>5.1). Associations between NLR, SYNTAX score, and clinical variables were analysed using univariable methods because the number of high SYNTAX cases and 30-day mortality events did not permit reliable multivariable modelling.

Results: The mean age of the cohort was 46.5±7.4 years, and 77.0% were male. Diabetes mellitus, hypertension, and smoking were present in 66, 67, and 74 percent of patients, respectively. The mean SYNTAX score was 15.6±9.1, and the mean NLR was 4.7±1.6. A moderate positive correlation was observed between NLR and SYNTAX score (r=0.48, p<0.001). SYNTAX scores increased progressively across NLR categories, with mean values of 11.27 in the low group, 15.0 in the intermediate group, and 17.79 in the high group. Only seven patients (7.0 %) had a SYNTAX score ≥33, which limits the precision of subgroup estimates. An NLR greater than 5.5 identified patients with SYNTAX ≥33 with 100% sensitivity, 77.4% specificity, a positive predictive value (PPV) of 75%, a negative predictive value (NPV) of 100%, and an area under the ROC curve of 0.88 (95% CI: 0.78-0.95). During the 30-day follow-up, four patients died. An NLR greater than 5.4 predicted 30-day mortality with 100% sensitivity, 72.9% specificity, and an AUC of 0.86 (95% CI: 0.71-0.97). Mortality-related estimates should be interpreted cautiously because of the small number of events.

Conclusions: In NSTE-ACS, NLR correlates with the anatomical complexity of coronary artery disease and may provide useful supplementary information during early risk assessment. However, the proposed threshold should be regarded as exploratory because of the limited number of patients with very high SYNTAX scores and the small number of mortality events. NLR should be considered a complementary marker rather than a replacement for established clinical risk stratification tools. Larger, multicentre studies with external validation are needed before NLR-based thresholds can be integrated into routine clinical pathways.

## Introduction

Myocardial infarction (MI) results from irreversible cardiomyocyte necrosis, most commonly triggered by atherosclerotic plaque rupture, endothelial erosion, or plaque fissuring. It may also arise from inflammatory, metabolic, or toxin-mediated myocardial injury [1]. Non-ST-segment elevation myocardial infarction (NSTEMI) and ST-segment elevation myocardial infarction (STEMI) differ in incidence and short-term outcomes. Contemporary registries show that NSTEMI has become more prevalent, accounting for approximately 75% of acute myocardial infarctions, whereas STEMI accounts for about 23% [2]. Although in-hospital mortality remains higher in STEMI (5.4% vs. 2.7% for NSTEMI), this difference diminishes with longer follow-up, and three-year mortality is comparable between the two groups [3].

Because patients with non-ST-elevation acute coronary syndrome (NSTE-ACS) often undergo an early but not immediate invasive strategy, unlike those with STEMI who receive emergent reperfusion as standard care, early risk stratification is particularly important in NSTE-ACS. A simple biomarker such as the neutrophil-to-lymphocyte ratio (NLR) could aid in prioritizing patients for invasive assessment [4].

Synergy between percutaneous coronary intervention with taxus and cardiac surgery (SYNTAX) score is an angiographic grading system used to evaluate the anatomical extent and complexity of coronary artery disease (CAD) [5]. While clinically useful, it has several limitations. SYNTAX scoring relies on visual interpretation of angiographic images and is therefore subject to inter and intra observer variability, particularly in complex lesions such as bifurcations and chronic total occlusions [6]. Scoring is also time-consuming and may not be feasible in high-volume catheterization laboratories [7]. Furthermore, the score has limited predictive value in certain patient populations, such as those with diabetes and multivessel disease, where it does not independently predict outcomes after coronary artery bypass grafting; thus, it should not be used as the sole determinant of revascularisation strategy [8].

Given these limitations, complementary biomarkers that capture the biological activity underlying plaque complexity may enhance risk stratification. The neutrophil-to-lymphocyte ratio (NLR), derived from routine complete blood counts, integrates two opposing arms of the immune response [9]. Neutrophils contribute to atherosclerotic progression and plaque destabilization through endothelial injury, release of proteolytic enzymes and matrix metalloproteinases, generation of reactive oxygen species, and formation of neutrophil extracellular traps, all of which promote fibrous-cap thinning and thrombogenicity. In contrast, lymphopenia reflects heightened physiological stress, cortisol-mediated immune suppression, and impaired adaptive immunity. Elevated NLR therefore represents a state of heightened systemic inflammation and immune dysregulation [10].

Numerous studies have linked higher NLR values with increased short- and long-term mortality in both general and cardiovascular populations, including patients with acute coronary syndromes [11]; however, the prognostic performance of NLR has been inconsistent, with heterogeneity in reported cut-off endpoints, and adjustment for confounders. Several studies have also demonstrated positive associations between NLR and angiographic disease severity, including correlations with SYNTAX score. Nevertheless, most data derive from single-centre cohorts with limited generalisability, and findings have not been uniformly consistent [12,13].

Evidence from the Middle East and North Africa regions with earlier onset of CAD and a high burden of traditional risk factors remains sparse. Understanding whether NLR correlates with angiographic complexity in these populations may be particularly relevant [14].

In this study, we examined the relationship between baseline NLR and SYNTAX score in an Egyptian NSTE-ACS cohort and explored potential NLR thresholds associated with complex CAD and short-term mortality. Because the SYNTAX score reflects structural coronary anatomy and the NLR reflects systemic inflammatory activity, we hypothesized that higher NLR would be associated with greater angiographic complexity and could provide incremental information to complement the anatomical assessment.

## Materials and methods

Study population

We prospectively included 100 consecutive patients with NSTE-ACS who underwent invasive coronary angiography at two tertiary care centres in Egypt between July 2020 and June 2021. NSTE-ACS was diagnosed according to the criteria outlined in the 2020 European Society of Cardiology (ESC) guidelines [12].

Exclusion criteria

Patients were excluded if they had persistent ST-segment elevation on ECG, clinical evidence of systemic infection, chronic obstructive pulmonary disease, established chronic inflammatory or autoimmune disease, active malignancy, or chronic steroid use.

Clinical assessment

All patients underwent standardised clinical evaluation. A detailed medical history was obtained, including presenting symptoms, cardiovascular risk factors (hypertension, diabetes mellitus, dyslipidaemia, smoking status, and body mass index), and additional comorbidities such as chronic kidney disease (CKD). A resting 12-lead ECG was performed for all patients. Routine laboratory investigations followed, and all patients subsequently underwent coronary angiography.

Laboratory measurements and timing

A complete blood panel, including full blood count, kidney function tests, lipid profile and cardiac biomarkers, was obtained once on admission, before coronary angiography. Sampling was not standardised relative to symptom onset. Laboratory analyses were performed at the two participating centres, both using the same automated haematology analyser and harmonised operating protocols. Although this approach reduced methodological variability, minor inter-laboratory differences cannot be fully excluded. There were no missing data for clinical, laboratory, or angiographic variables, and all analyses were performed using complete-case data.

Angiographic assessment

Coronary angiograms were scored using the SYNTAX system. Two experienced interventional cardiologists (each with >5 years of coronary intervention experience), blinded to all clinical and laboratory data, independently calculated SYNTAX scores from the clinical and laboratory data using the online calculator available at www.syntaxscore.org [15]. Discrepancies of ≥5 points were resolved through consensus discussion. Formal interobserver agreement statistics were not calculated.

Outcome measurement

Thirty-day all-cause mortality was determined through review of hospital electronic medical records. No patients were lost to follow-up at 30 days.

Variable classification

In this study, patients were stratified into tertiles based on admission NLR: low NLR (≤3.65), intermediate NLR (>3.65-≤5.1), and high NLR (>5.1). Patients were also subdivided by SYNTAX score into three categories: low (≤22), intermediate (23-32), and high (≥33) according to the original SYNTAX trial classification [5].

Statistical analysis

All analyses were conducted using SPSS Statistics (IBM Corp., 2011, IBM SPSS Statistics for Windows, Version 20). The distribution of continuous variables was assessed using the Shapiro-Wilk test and visual inspection of histograms and Q-Q plots. Normally distributed variables were summarised as mean±standard deviation and compared using the independent samples t-test. Non-normally distributed variables were presented as median (interquartile range) and compared using the Mann-Whitney U test. Categorical variables were compared using the Chi-square test or Fisher’s exact test when appropriate.

Correlations between continuous variables were assessed using Pearson’s correlation coefficient for approximately normally distributed variables and Spearman’s rank correlation otherwise. A p-value < 0.05 was considered statistically significant.

A priori sample size calculation was not performed. The sample consisted of all consecutive eligible patients during the study period. The number of high-SYNTAX cases and 30-day deaths was insufficient for reliable multivariable modelling, and therefore all analyses were unadjusted and exploratory.

Receiver operating characteristic (ROC) curve analysis was performed to determine the optimal NLR cut-off for predicting high SYNTAX score and 30-day mortality. Area under the curve (AUC) values with 95% confidence intervals were reported. ROC-derived cut-off values were not internally validated (e.g., by bootstrapping or cross-validation); the thresholds identified should therefore be considered exploratory and may be optimistic.

Sensitivity analyses were conducted by repeating the primary analyses after excluding patients with extreme NLR values (top 5% of the distribution) and by stratifying results according to diabetes mellitus and CKD status. These analyses were exploratory and aimed to assess the robustness of the observed associations.

Ethical considerations

The study design, eligibility criteria, and primary analytical plan, including correlation analyses and exploratory ROC evaluation of high SYNTAX score and 30-day mortality, were prespecified in the protocol approved by the institutional ethics committee. Ethical approval was obtained from the Research Ethics Committee of Beni-Suef University (Approval number: FMBSUREC/03092019/Narouz). Written informed consent was obtained from all participants.

## Results

The study included 100 patients. The mean age was 46.5±7.4 years; 77 (77.0%) were male, and 23 (23.0%) were female. The remaining baseline characteristics, risk factors, mortality outcomes, and patient categories stratified by NLR and SYNTAX score are presented in Table 1.

**Table 1:** Baseline characteristics and risk factors in the studied population. Continuous variables are reported as mean±SD. Shapiro-Wilk testing and visual inspection of histograms and Q-Q plots indicated non-normal distribution for cholesterol, LDL-C, triglycerides, CK, and CK-MB; however, mean±SD is presented for consistency and comparability with prior literature. Categorical variables are presented as n (%). CK: creatine kinase; CK-MB: creatine kinase-MB isoenzyme; HDL-C: high-density lipoprotein cholesterol; hs-cTnI: high-sensitivity cardiac troponin I; LDL-C: low-density lipoprotein cholesterol; NLR: neutrophil-to-lymphocyte ratio; TLC: total leukocyte count, SYNTAX: synergy between percutaneous coronary intervention with taxus and cardiac surgery.

Variables	N=100 (%)
Age (years)	46.53±7.43
Male gender	77 (77.0%)
Female gender	23 (23.0%)
Diabetes mellitus	66 (66.0%)
Hypertension	67 (67.0%)
Smoking	74 (74.0%)
Lipid profile	
Cholesterol (mg/dL)	216.87±38.93
LDL-C (mg/dL)	145.78±29.00
HDL-C (mg/dL)	33.74±7.02
Triglycerides (mg/dL)	118.31±39.57
Kidney functions	
Urea (mg/dL)	33.93±21.21
Creatinine (mg/dL)	0.98±0.21
Cardiac biomarkers	
CK (U/L)	385.20±149.69
CK-MB (U/L)	39.58±13.22
hs-cTnI (ng/ml)	0.018±0.012
TLC (×10⁹/L)	8.81±4.30
NLR	4.78±1.61
SYNTAX score	15.61±9.14
Mortality	4 (4.0%)

With respect to angiographic complexity, 37 patients (37.0%) had a low SYNTAX score (≤22), 56 (56.0%) had an intermediate score (23-32), and 7 (7.0%) had a high score (≥33). The small number of patients with high SYNTAX scores may limit the precision of estimates derived for this subgroup.

Correlation analysis

NLR Correlations

NLR showed statistically significant positive correlations with age, total cholesterol, LDL-C, urea, creatinine, CK, CK-MB, TLC, hs-cTnI, and the SYNTAX score, as shown in Table 2. In contrast, correlations with BMI, HDL-C, and triglycerides were not statistically significant. Notably, the correlation between NLR and SYNTAX score was of moderate magnitude (r=0.481; 95% CI: 0.31-0.62; p<0.001).

**Table 2:** Correlation between NLR with baseline characteristics. CK: creatine kinase, CK-MB: creatine kinase-MB isoenzyme; HDL-C: high-density lipoprotein cholesterol; hs-cTnI: high-sensitivity cardiac troponin I; LDL-C: low-density lipoprotein cholesterol; NLR: neutrophil-to-lymphocyte ratio; TLC: total leukocyte count, SYNTAX: synergy between percutaneous coronary intervention with taxus and cardiac surgery.

Variables	NLR	
	R	p-value
Age	0.242	0.019
BMI (kg/m²)	0.044	0.664
Cholesterol (mg\dL)	0.213	0.033
LDL-C (mg\dL)	0.423	<0.001
HDL-C (mg/dL)	-0.086	0.395
Triglycerides (mg/dL)	0.022	0.831
Urea (mg/dL)	0.395	<0.001
Creatinine (mg/dL)	0.379	<0.001
CK (U/L)	0.289	0.004
CK-MB( U/L)	0.441	<0.001
TLC (×10⁹/L)	0.241	0.016
SYNTAX score	0.481	<0.001
hs-cTnI (ng/ml)	0.244	0.015

We applied the Benjamini-Hochberg procedure to reduce type I error arising from multiple correlations; the associations between NLR and LDL-C, urea, creatinine, CK-MB and SYNTAX score remained significant after correction. All correlations reported were unadjusted. Because of the modest sample size and number of outcome events, we did not perform multivariable modelling to adjust for potential confounders, and the correlation analyses should be interpreted as exploratory.

NLR Categories Correlation

The distribution of NLR within each category was as follows: low NLR group, median 3.0 (IQR: 2.4-3.5); intermediate NLR group, median 4.3 (IQR: 3.9-4.8); and high NLR group, median 6.1 (IQR: 5.6-7.2).

On comparing the three NLR groups, the mean age was 49.4 in the high group vs 46.4 in the intermediate group vs 43.7 in the low group (p=0.007). The incidence of diabetes, value of TLC, and hs-Troponin (ng/dL) were significantly higher in the high NLR group.

Similarly, a significant difference in mean SYNTAX scores was observed across the NLR groups, with values of 17.7, 15.0, and 11.27 in the high, intermediate, and low groups, respectively (p<0.001). The comparative data are shown in Table 3.

**Table 3:** Comparison between low NLR, intermediate NLR and high NLR regarding baseline characteristics. *: Chi-square test; •: Kruskal-Wallis test. Categorical variables were compared using the Chi-square test, and continuous variables across NLR categories were analysed using the Kruskal–Wallis test. These comparisons are unadjusted and should be regarded as exploratory. CK: creatine kinase, CK-MB: creatine kinase-MB isoenzyme; HDL-C: high-density lipoprotein cholesterol; hs-cTnI: high-sensitivity cardiac troponin I; LDL-C: low-density lipoprotein cholesterol; NLR: neutrophil-to-lymphocyte ratio; TLC: total leukocyte count, SYNTAX: synergy between percutaneous coronary intervention with taxus and cardiac surgery.

Variables		Low NLR	Intermediate NLR	High NLR	Test value	p-value
		n=33	n=33	n=34		
Age (Mean±SD)		43.75 ± 6.64	46.45±7.51	49.47±7.22	5.234•	0.007
Sex (n, %)	Male	27 (81.8%)	26 (78.8%)	24 (70.6%)	1.281*	0.527
	Female	6 (18.2%)	7 (21.2%)	10 (29.4%)		
BMI (Mean±SD)		29.24±5.41	27.54±3.57	28.62±3.45	1.359•	0.262
Diabetes (n, %)	No	19 (57.6%)	8 (24.2%)	7 (20.6%)	12.299*	0.002
	Yes	14 (42.4%)	25 (75.8%)	27 (79.4%)		
Hypertension (n, %)	No	12 (36.4%)	12 (36.4%)	9 (26.5%)	0.993*	0.609
	Yes	21 (63.6%)	21 (63.6%)	25 (73.5%)		
Smoking (n, %)	No	8 (24.2%)	6 (18.2%)	12 (35.3%)	2.628*	0.269
	Yes	25 (75.8%)	27 (81.8%)	22 (64.7%)		
Cholesterol (mg/dL)		226.12±46.73	211.24±32.50	213.35±35.73	1.427	0.245
LDL (mg/dL)		152.24±30.09	138.88±26.14	146.21±29.86	1.786	0.173
HDL (mg/dL)		34.62±7.36	34.36±7.49	32.26±6.14	1.143	0.323
Triglycerides (mg/dL)		116.82±38.27	119.73±42.54	118.38±38.96	0.044	0.957
Urea (mg/dL)		29.87±18.44	28.16±21.51	43.47±20.68	5.771	0.004
Creatinine (mg/dL)		0.95±0.22	0.98±0.19	1.00±0.21	0.578	0.563
CK (U/L)		338.84±134.53	369.73±138.74	455.51±157.81	4.839	0.010
CK-MB (U/L)		34.36±12.77	39.09±13.87	45.11±10.98	6.152	0.003
TLC (×10⁹/L)		6.51±3.96	9.18±4.16	10.69±3.79	9.506	<0.001
SYNTAX score		11.27±6.49	15.0±9.10	17.79±11.82	4.033	<0.001
hs-cTnI (ng/ml)		0.013±0.011	0.014 ± 0.008	0.027±0.012	17.59	<0.001

ROC analysis for 30-day mortality

**Figure 1:**
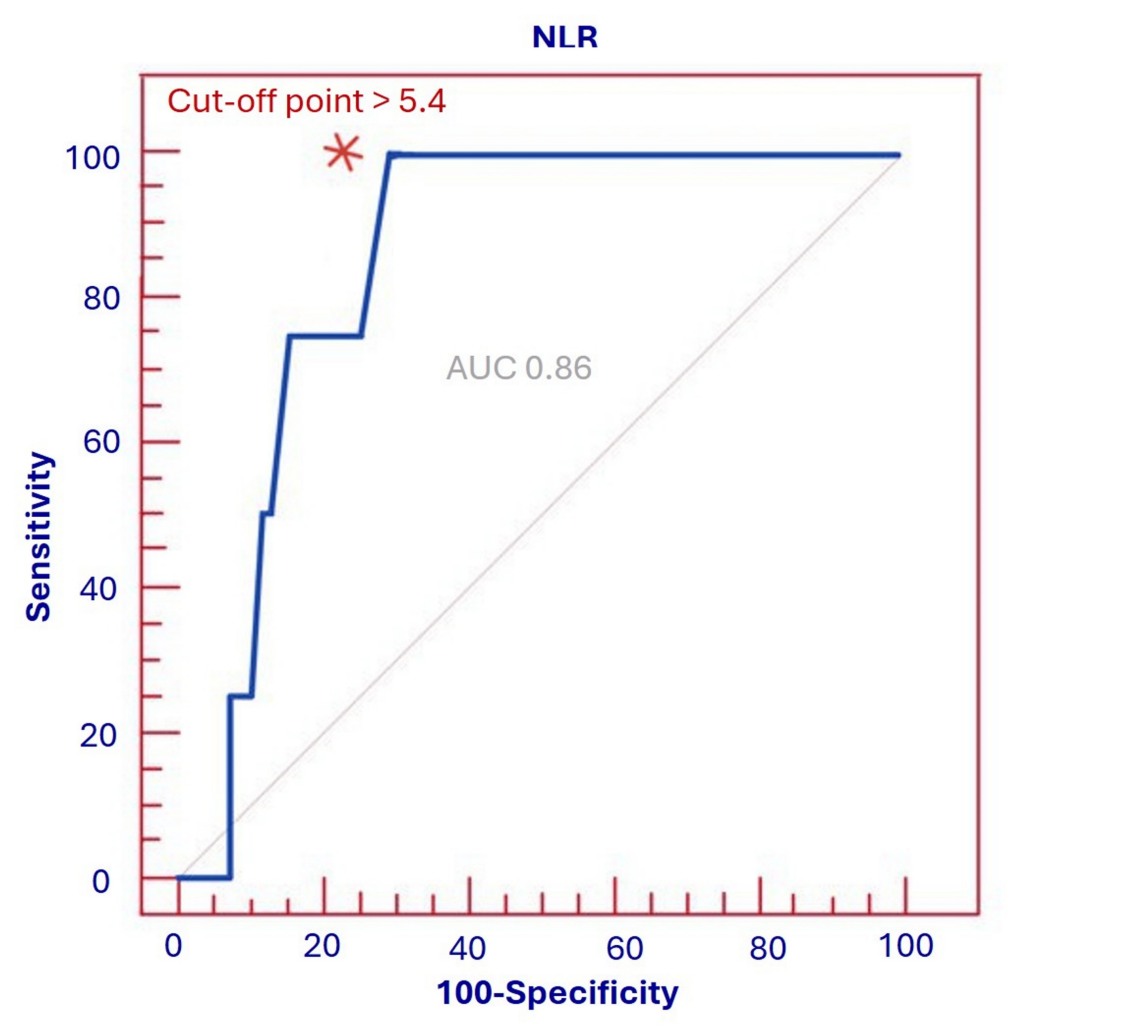
ROC curve of NLR to predict 30-day mortality. The asterisk mark (*) indicates the best cut-off value of NLR>5.4, selected based on the highest combination of sensitivity and specificity. The area under the curve (AUC) was 0.86, indicating good discriminative ability of NLR for predicting short-term mortality.

During the 30-day follow-up, four patients (4.0%) died. ROC analysis was performed to estimate the optimal NLR threshold for predicting 30-day mortality. An NLR value >5.4 was associated with a sensitivity of 100%, specificity of 72.9%, overall accuracy of 86%, a positive predictive value (PPV) of 73.3% and a negative predictive value (NPV) of 100%. The corresponding ROC curve is shown in Figure 1. The area under the curve (AUC) was 0.86 (95% CI 0.71-0.97), indicating good discriminative ability of NLR for short-term mortality. Given that the ROC analysis is based on only four deaths, these estimates should be interpreted cautiously and considered hypothesis-generating.

ROC analysis for high SYNTAX score

**Figure 2:**
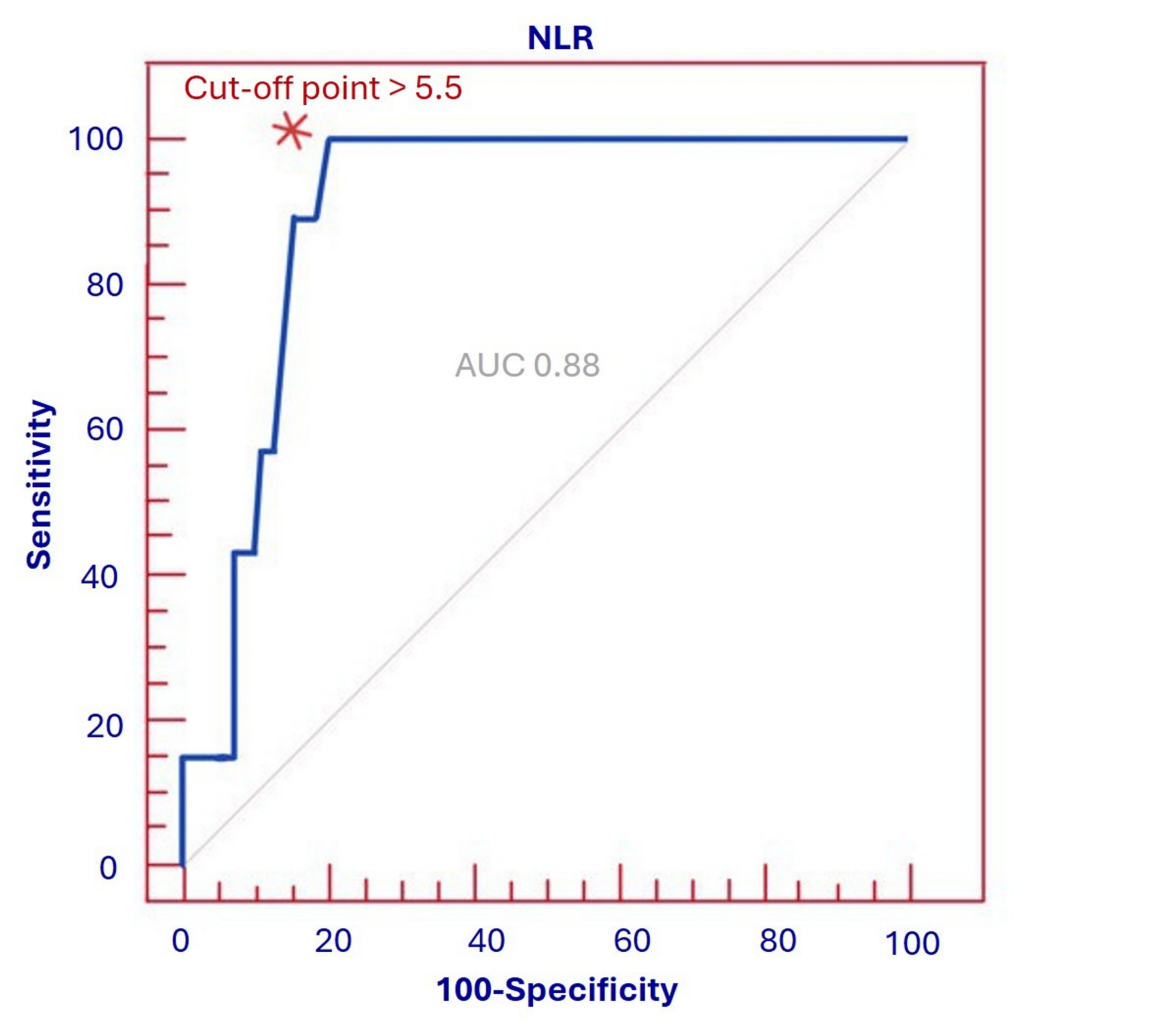
ROC curve of NLR to detect SYNTAX score. The asterisk mark (*) indicates the best cut-off value of NLR>5.5, selected based on the highest combination of sensitivity and specificity. The area under the curve (AUC) was 0.88, suggesting strong diagnostic performance in detecting angiographically complex coronary artery disease.

ROC assessment was also conducted to estimate the best NLR threshold for identifying patients with a high SYNTAX score (≥33). An NLR >5.5 yielded a sensitivity of 100.0%, specificity of 77.4%, PPV of 75.0%, NPV of 100.0% and overall accuracy of 88% for detecting high SYNTAX scores. The ROC curve for this analysis is presented in Figure 2. The AUC was 0.88 (95% CI: 0.78-0.95), suggesting good discriminative performance of NLR for angiographically complex CAD. However, given the very small number of patients with SYNTAX ≥33 (n=7), the apparent 100% sensitivity and NPV are likely overestimates and should be interpreted with particular caution. The ROC-derived cut-off values were not internally validated and should therefore be regarded as exploratory.

## Discussion

Inflammation plays a central role in the initiation, progression, and complications of atherosclerotic disease. The NLR, a readily available marker derived from routine blood counts, has emerged as an index of systemic inflammatory activation and has shown prognostic value across several cardiovascular settings [16]. From a biological standpoint, an elevated NLR reflects a pro-inflammatory and pro-thrombotic state that may predispose to diffuse and complex coronary atherosclerosis. Neutrophils promote endothelial injury, oxidative stress, and release proteolytic enzymes and matrix metalloproteinases, while also forming neutrophil extracellular traps that facilitate thrombus formation. In parallel, lymphopenia reflects physiological stress and impaired adaptive immunity. Together, these shifts yield a heightened NLR that integrates amplified innate activation with relative suppression of adaptive regulatory mechanisms, a pattern that aligns with the presence of extensive and morphologically complex coronary disease, as captured by the SYNTAX score [10].

Previous studies have demonstrated associations between NLR and coronary artery disease severity in STEMI and chronic coronary syndrome [16], although data specific to NSTE-ACS have been less consistent. In our cohort, NLR showed a moderate correlation with SYNTAX score (r=0.48), which is comparable to or slightly higher than correlations reported for NLR and other inflammatory markers with angiographic indices in ACS and chronic CAD populations, where coefficients typically range from 0.30 to 0.50. This magnitude suggests that NLR provides meaningful anatomical discrimination and may serve as a surrogate marker of lesion complexity [17].

Our ROC analysis demonstrated that an NLR>5.5 identified patients with high SYNTAX scores with a sensitivity of 100% and specificity of 77.4%, while an NLR>5.4 predicted 30-day mortality with similarly good discriminative performance. To our knowledge, no prior studies have proposed an NLR cut-off for detecting high SYNTAX scores in NSTE-ACS.

The NLR cut-off identified in our cohort (>5.5) is broadly comparable to thresholds reported in previous ACS studies. For example, Soylu et al. found that an NLR of 4.5 predicted in-hospital mortality with an AUC of 0.86 [17], while a meta-analysis of ACS populations suggested that values around 5.0 may represent a clinically meaningful risk threshold [18]. Our slightly higher threshold may reflect population-level characteristics, as our cohort consisted exclusively of Egyptian patients, who typically present with earlier-onset coronary artery disease and a higher prevalence of traditional cardiovascular risk factors. Such demographic and cardiometabolic differences may plausibly shift inflammatory baselines and alter NLR distributions. Therefore, the threshold identified here may hold specific relevance for Middle Eastern and North African populations, although validation in larger regional cohorts is required.

Nevertheless, several limitations temper the diagnostic implications of our findings. Only seven patients in our study had very high SYNTAX scores (≥33), limiting the precision of ROC-derived estimates for complex disease. The apparently perfect sensitivity and high negative predictive value are therefore likely inflated due to sparse data at the extreme end of angiographic complexity and should be viewed as exploratory rather than definitive. Although this study included patients from two tertiary hospitals, the dataset functioned analytically as a single pooled cohort. The ROC thresholds were not internally validated (e.g., via bootstrapping) nor tested in an external population, raising the possibility of optimism bias in the reported diagnostic metrics.

We also observed that patients in the high NLR group more frequently had diabetes mellitus, higher creatinine values, elevated TLC, and higher hs-cTnI concentrations. These comorbid and infarct-related factors are independently associated with worse outcomes and more advanced CAD, and may partially account for the observed relationship between NLR and SYNTAX score [19]. Because our analyses were unadjusted and we did not perform multivariable regression due to the modest sample size and the limited number of events, we cannot determine whether NLR is an independent marker of angiographic complexity or reflects the cumulative burden of renal dysfunction, metabolic disease, and myocardial injury. Larger studies with adequate event numbers should evaluate whether NLR provides incremental predictive value beyond established clinical and biochemical predictors.

Another important consideration in our study is initial blood samples timing. NLR was measured once at admission, without standardisation to symptom onset. NLR can vary substantially within the first 24-48 hours of an ACS due to the evolving inflammatory response and degree of myocardial injury [20]. The lack of uniform timing may therefore introduce variability that either attenuates or distorts true associations between inflammatory activation and lesion complexity. This study showed a predominantly male distribution, which may limit generalisability, and sex-specific analyses were not performed.

The clinical interpretation of our findings requires a careful evaluation. The pattern of high sensitivity and negative predictive value but only modest specificity suggests that NLR is better suited as a rule-out tool rather than a stand-alone selector for invasive management. An NLR below the proposed threshold may help identify patients unlikely to harbour highly complex coronary anatomy or to experience early mortality, whereas an elevated NLR should be interpreted alongside clinical evaluation, cardiac biomarkers, and established risk scores. Our data do not imply that NLR should replace validated systems such as GRACE or TIMI. Prior work has shown that NLR correlates with GRACE and that combining inflammatory markers with conventional scores may enhance prognostic accuracy [17]. Future research should examine whether integrating NLR into multivariable models or established risk tools improves discrimination and reclassification in NSTE-ACS.

Several studies have linked the NLR value to the prognosis of acute coronary syndrome. In one study, patients with ACS had a worse prognosis with a higher NLR score [21]. Similarly, a higher NLR was associated with an increased incidence of clinical adverse events within one year after coronary interventions in individuals with NSTE-ACS [22]. Lin et al., consistent with our findings, suggested that NLR is a useful marker for predicting cardiovascular mortality after percutaneous coronary intervention in patients presenting with STEMI [23]. These findings align with our observation that higher NLR values were associated with both anatomical complexity and short-term mortality. However, our study is limited by its modest sample size of 100 patients, the low number of deaths during the 30-day follow-up, and the absence of long-term outcome data. Because only individuals with NSTE-ACS were included, our findings and the proposed NLR threshold may not apply to the broader ACS population. Additionally, the interval from symptom onset to presentation was not recorded, which may have influenced baseline inflammatory measurements. Nevertheless, selection bias is unlikely given the consecutive recruitment of eligible patients.

While the SYNTAX score provides a structured angiographic assessment of lesion complexity with established prognostic value after revascularisation, it is limited by inter-observer variability and its time-intensive calculation. In contrast, the NLR is inexpensive, widely available, and rapidly obtained through routine haematology testing. Further studies are required to evaluate whether the NLR threshold identified here is reproducible and whether NLR can reliably predict coronary artery disease severity and early mortality at admission. Larger prospective multicentre studies are necessary to clarify the role of NLR in risk stratification among patients with NSTE-ACS.

## Conclusions

In patients with NSTE-ACS, the neutrophil-to-lymphocyte ratio (NLR) appears to reflect the underlying burden and complexity of coronary artery disease. Its clinical appeal stems from its simplicity, wide availability, and ability to provide additional information during early risk assessment. Nonetheless, the NLR threshold identified in this study (>5.5) should be interpreted as exploratory. Its reliability is limited by the small number of patients with very high SYNTAX scores, which affects the stability of the ROC-derived performance estimates.

NLR should therefore be considered a complementary marker rather than a replacement for established clinical risk stratification tools such as the GRACE or TIMI scores. Before NLR can be meaningfully incorporated into diagnostic or therapeutic pathways, the proposed cut-off requires validation in larger, multicentre studies and across diverse populations and healthcare settings. Although the current findings are encouraging, they highlight the need for more comprehensive and methodologically rigorous research to determine whether NLR-based thresholds can be used confidently in routine clinical practice.
